# Supplementary figures and images for: Social factors associated with reversing frailty progression in community-dwelling late-stage elderly people: An observational study
Source: PLoS One. 2021 Mar 3;16(3):e0247296. doi: 10.1371/journal.pone.0247296 (PMC7928521; doi:10.1371/journal.pone.0247296)

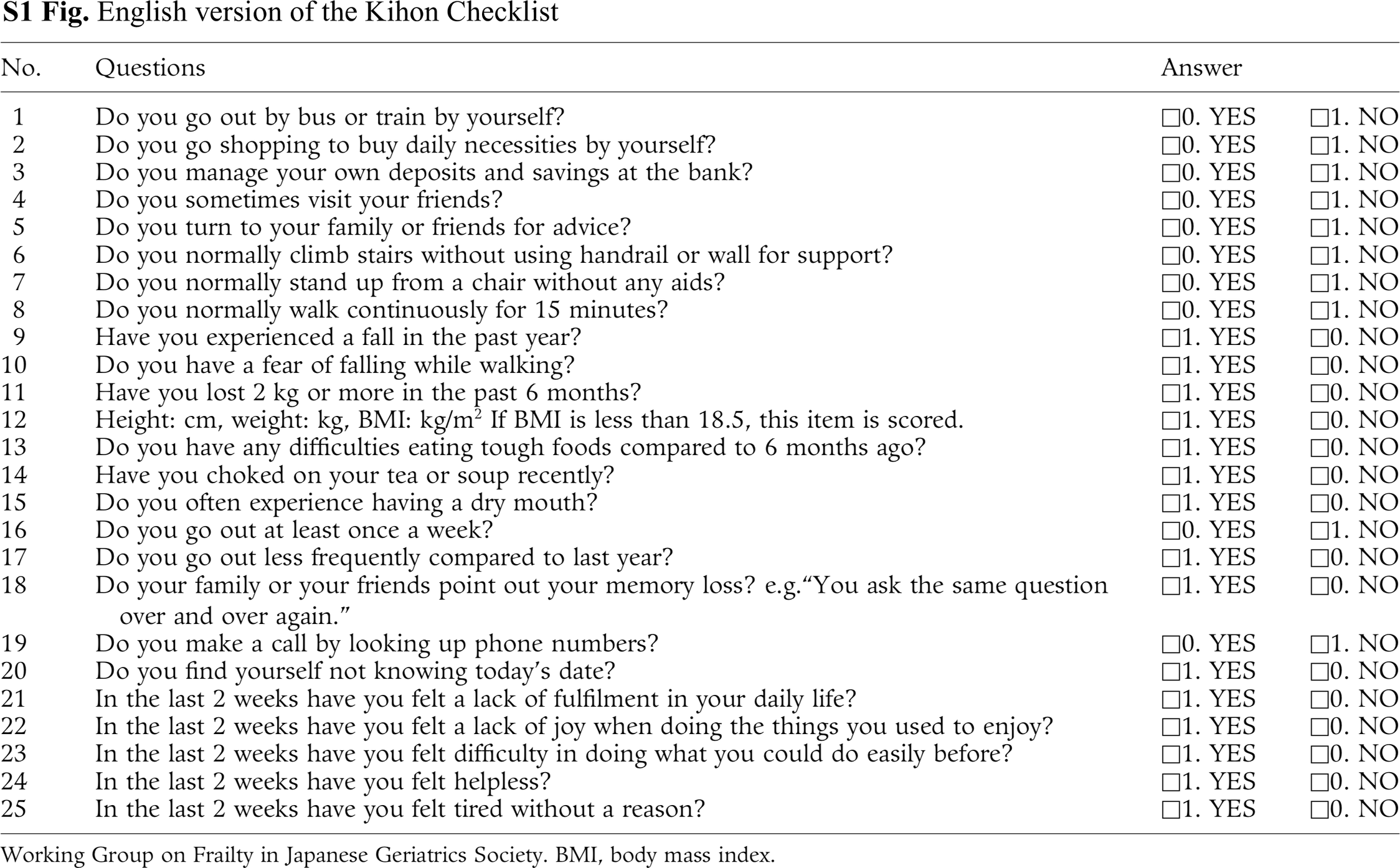

Supplement: S1 Fig — (TIF) [file pone.0247296.s001.tif]
