# Supplementary material for: Social factors associated with reversing frailty progression in community-dwelling late-stage elderly people: An observational study
Source: PLoS One. 2021 Mar 3;16(3):e0247296. doi: 10.1371/journal.pone.0247296 (PMC7928521; doi:10.1371/journal.pone.0247296)
Supplement: Attachment — Submitted filename: Response to Reviewers.docx [file pone.0247296.s002.docx]

**Authors' Response to Reviewers**

**For Reviewer #1**

Thank you for your valuable comments and your appreciation of the content of our manuscript. We have replied to your comments point-by-point as shown below.

We have made general corrections to the manuscript, including revisions to the data analysis. We also reconfirmed the STROBE checklist, changed the title, specified the data collection period, and corrected inappropriate terms.

**Introduction**

- *the presented definition of frailty is not one that is commonly used in the field and seminal pieces of work on frailty (such as those by Fried and Rockwood) are not used in the definition discussion. What is described is far from what would be considered the current characterization of frailty and the line of reasoning presented to explain their description is hard to follow.*
- The frailty judgment method using the KCL has validity with the CHS criteria and Frailty index, based on the concept of Fried et al. (P4 L86-94).
- *"escape from frailty" is not terminology I would use. Instead, you are discussing reversing frailty progression. This becomes much easier to comprehend and more intuitively also describes a transition from frailty to pre-frailty.*
- In the entire paper, the inappropriate term "escape" has been changed to "recovery" or "reversibility". The title of the paper has also been changed.
- *subjective age was presented in the introduction but not described until much later. A better descriptor of what the authors were actually discussing is a 'younger subjective age' or similar that would better represent what is being discussed.*
- The background and reasons for including subjective age in the evaluation items have been explained by presenting the results of previous studies in the "Methods" section (P7 L186-P8 L191).
- *As a less used measure of frailty, providing more data on KCL correlation to more widely accepted methods would be recommended.*
- We have stated that KCL is an evaluation listed in the Frailty Management Guidelines for the Asia-Pacific region (P4 L90-94).
- *“senile decay”??? (line 89) – better description of why reversing or slowing frailty progression is important*
  - We modified this sentence as suggested (P4 L95).
- *There have been a number of intervention studies that could have been discussed in the background literature including best recommendations thus far. Only one was mentioned – this was not an extensive literature review by any means.*
- We cited a new systematic review of the effects of interventions on frailty and added the level of evidence for interventions that have been reported to be effective (P4 L96-107).
- *Clearer description needed of “factors that assist” in reversing frailty and distinguishing those from “characteristics" of those who see frailty reversibility.*
- *No hypothesis is presented.*
- We clarified the research hypothesis and separately described factors necessary to recover from frailty and the social activity factors of those who recovered (P5 L108-113).

**Methods**

- *How was the study population found?*
- We used data from a complete enumeration of the elderly living in the community of Ikoma City, Nara Prefecture, Japan, who have not been certified as requiring long-term care by the long-term care insurance system.
- *Flow chart of participant progression was unclear. “Certified”? – how was ‘certification’ determined. Flow chart talks about insurance? Why would participants be dropped if they now required LTCI through the follow up period? I thought this was the authors' definition of frailty, albeit one that I disagree with.*
- We have clarified the term for "certification". In addition, from the viewpoint of care prevention, this study explained that the follow-up survey excluded those who were certified as requiring care by the government (i.e., deterioration from frailty to needing care) (P5 L123-127).
- *Frailty 'escapers' is not well described. Instead what is being described is reversing frailty progression.*
- We have clarified the definition of those who have recovered from frailty, and have deleted the expression "escape".
- *Examining those who did not have their frailty progress would also have been interesting.*
  - As you pointed out, it is necessary to consider "maintenance" of the frailty state, but since this study focuses on "improvement" from the frailty, it is not included in the analysis. We will make it a future goal.

**Results**

- *Describe sex but use term “women” – women refers to gender, female refers to sex*
- We have revised the terms throughout the paper.
- *Is ‘escape from frailty’ just those who went from frail to robust or also those who went from pre-frail to robust? This seems to only be described in the discussion.*
- We have clarified the definition of those who have recovered from frailty (P8 L211-212).
- *I would recommend presenting change in frailty status differently graphically and statistically. With a checklist with many variables like the KCL, this data could have been presented in a continuous fashion as opposed to categorical changes which result in a loss of information.*
  - In addition to Fig. 1, we created a new crosstabulation table to clarify the change in the frailty state. Results of the χ-square test and the residual test analyses were added.

**Discussions**

- *Definition of 'escape from frailty' only provided in the discussion?*
  - Throughout the paper, the expression "escape from frailty" was deleted and corrected to "recovery from frailty" or "participants who recovered from frailty".
- *Authors start on good discussion of relative reversibility of frail vs pre-frail states but don’t follow through on description of why this might be or references to seminal works.*
  - We have made major revisions to the first half of the discussion. In addition, we added the part related to the reversibility of frailty and explained the importance of social frailty by citing the results of previous research (P14 L283-304).
- *What do we do with this information? Authors do not provide recommendations for how to incorporate findings into any kind of practice.*
- Based on the results of this research, we have clarified specific counterplans that are considered important for promoting care prevention in Japan (P17 L371-381).
- *Conclusions have to be more strongly discussed as being factors associated with reversing frailty as this assessment is not an intervention.*
- We have added more specific recommendations to our conclusions.

**For Reviewer #2**

Thank you for your very valuable comments and your appreciation of the content of the paper. We have replied to your comments point-by-point as shown below.

**Major comments:**

- *The authors collect disease burden in the present study, which appear to not be a part of their frailty measure. How might an individual’s disease burden affect their “escape” from frailty?*
- We added a consideration of why the disease burden was evaluated as a variable and that it did not independently affect recovery from frailty (P16 L353-357).
- *Here the authors only assess “escape from frailty” However it would be useful to determine if there is worsening frailty among the group, and which factors might lead to that.*
- As you pointed out, it is important to describe the deterioration from frailty, but the deterioration from frailty indicates that long-term care insurance services are required. This may include the development of serious illnesses such as stroke. Therefore, we focused on factors that are important in improvement from weakness in this study.
- *It would be interesting to determine the cumulative effect of factors examined here on escape from and transition to frailty (and those who stay stable). For example, do the number of factors an individual has have a greater odds of transitioning to a better state?*
- As an additional analysis, we analyzed the effect of the number of factors on recovery from frailty and added it to the results.

**Minor comments:**

- *Please include in the abstract the follow up time period (median preferable)*
- We have added a median follow-up period (P5 L123). The survey is conducted in a fixed month every year, and there is almost no variation.
- *Please include in the abstract more detail about the Kihon checklist (i.e., how many items, range scores, what indicates frailty)*
- We have provided more detailed information in the abstract.
- *Title is misleading regarding “factors” that assist escape from frailty. It appears that the authors focus on specific factors which are predominantly social factors – this needs to be made clear*
- We have changed the title of the paper.
- *I think it would be useful to re review the manuscript for English language grammar/spelling/comprehension.*
- This time, we asked the company to proofread the English text more carefully. Thank you very much.
- *Please provide additional information on how participants were selected for receiving a survey. Was this a randomized process? Convenience sampling?*
- We have used data from a complete enumeration of the elderly living in the Ikoma city who have not been certified as requiring long-term care by the long-term care insurance system.
