# Supplementary material for: Social factors associated with reversing frailty progression in community-dwelling late-stage elderly people: An observational study
Source: PLoS One. 2021 Mar 3;16(3):e0247296. doi: 10.1371/journal.pone.0247296 (PMC7928521; doi:10.1371/journal.pone.0247296)
Supplement: Attachment — Submitted filename: Factors that assist escape from frailty (Takatori et al) - Nov 2020.docx [file pone.0247296.s003.docx]

Social factors that reverse frailty progression in community-dwelling late-stage elderly people: An observational study

Takatori et al. describe an observational cohort study over a 2-year period examining healthy lifestyle habits and participant characteristics that are associated with reversing frailty progression in Ikoma city, Japan. Thank you to the authors for addressing the suggested revisions in their manuscript. The authors addressed the suggested revisions, but the manuscript still requires work to improve clarity. The manuscript also requires thorough review of the English throughout for grammar and clarity.

- The title is much more descriptive of the article now but the way it is currently written implies a causative finding despite the identification of it being an observational study. Please edit the title so that it implies associations as the findings.
- It would be beneficial to review the English throughout the publication for grammar and clarity.

Abstract

- Line 29: Are those in nursing care not frail? The authors’ current first sentence would imply this, but this is not true. Based on the description from the Japanese Geriatric Society, frailty status can be associated with adverse outcomes like resorting to nursing care, but the description of frailty as being a state of health vulnerability as described late in the introduction is much more appropriate for use in the opening of the abstract than what is currently written.
- Line 42: It would be easier for the reader to interpret participant prevalence here by listing the recovery rate as a percentage of those who actually had frailty. Those who did not have frailty at baseline could not possible have reversed their frailty status.
- Line 49: Results presented should not be discussed as causative finding. This was an observational study. These should be identified as associations.

Introduction

- Again, the statement from the Japanese Geriatric Society is not being interpreted correctly in lines 60 and 61. They say it is vulnerability that leads to adverse outcomes such as need for nursing care. It would be incorrect to then imply that those receiving nursing care are not frail. That is just one of the adverse outcomes associated with frailty due to the health vulnerability it is characterized by.
- Line 64: Please provide a citation to support this change in demographic trend.
- Line 71: The end of this sentence should say “…were pre-frail.”
- Line 76: This is an interesting citation to select. There are a number of more recent consensus statements regarding frailty.
- Line 79: I do not think it is apt to say that one would diagnose frailty. Frailty is not a disease and not a commonly identified outcome in clinical practice, thus it would be more appropriate to say ”…for frailty identification.”
- Line 82-83: Accumulation of Deficits Models
- Line 85: “Frailty assessment” instead of “judgement”
- Lines 94-97: It is unclear what the authors are trying to state and the English here could use a thorough review.
- Line 93-94: The English in these two sentences needs to be reviewed. It is difficult to understand what this sentence is trying to present. Additionally, no citations are provided to support the statement made.
- Line 98-99: Citation required for this statement.
- Line 100-101: Please provide citations associated with the statements made here.
- Line 101-102: What reports have identified these factors? What factors were identified? Please provide citations here as well.
- Line 108: “…in improving frailty status”
- Lines 108-111: This sentence needs to be rewritten for clarity.

Methods

- Line 142; …frailty assessment

Results

- Fig 2: The English use here could use some review. “stayed robust”, “stayed pre-frail”, “remained frail”

Discussion

- Line 284: No citations provided to support this statement.
- Line 287: No citations provided to support this statement.
- Line 295: “No-frailty people”?
- Line 312-317: A lot of this text was already included in the results section. Please make this more succinct and focused on discussion aspects.
- Line 337-338: Unclear what this sentence is stating.
- Line 342: Please provide citations to support this statement.

Conclusions

- Again, these findings are stated quite strongly for an observational study. It should be clear that these findings are associations.
