# Supplementary material for: Social factors associated with reversing frailty progression in community-dwelling late-stage elderly people: An observational study
Source: PLoS One. 2021 Mar 3;16(3):e0247296. doi: 10.1371/journal.pone.0247296 (PMC7928521; doi:10.1371/journal.pone.0247296)
Supplement: Attachment — Submitted filename: Response to reviewer R2.docx [file pone.0247296.s004.docx]

**Authors' Response to Reviewers**

**For Reviewer #1**

Thank you for your valuable comments and your appreciation of the content of our manuscript. We have replied to your comments point-by-point below.

This time, we asked a proofreader to review the English expression and grammar in general, and revised it to improve the clarity of the content.

**Title**

- *The title is much more descriptive of the article now but the way it is currently written implies a causative finding despite the identification of it being an observational study. Please edit the title so that it implies associations as the findings.*
- We changed the wording that suggests a causal relationship and revised the title.

**Abstract**

- *Line 29: Are those in nursing care not frail?*
- *The authors’ current first sentence would imply this, but this is not true. Based on the description from the Japanese Geriatric Society, frailty status can be associated with adverse outcomes like resorting to nursing care, but the description of frailty as being a state of health vulnerability as described late in the introduction is much more appropriate for use in the opening of the abstract than what is currently written.*
- We have modified the wording about the definition of frailty at the beginning.
- *Line 42: It would be easier for the reader to interpret participant prevalence here by listing the recovery rate as a percentage of those who actually had frailty.*
- *Those who did not have frailty at baseline could not possible have reversed their frailty status.*
- We have re-calculated the recovery rate of the number of participants that have been classified as frailty at baseline as the denominator.
- *Line 49: Results presented should not be discussed as causative finding. This was an observational study. These should be identified as associations.*
  - We have changed the wording about causality (Line 51).

**Introduction**

- *Again, the statement from the Japanese Geriatric Society is not being interpreted correctly in lines 60 and 61.*
- *They say it is vulnerability that leads to adverse outcomes such as need for nursing care. It would be incorrect to then imply that those receiving nursing care are not frail.*
- *That is just one of the adverse outcomes associated with frailty due to the health vulnerability it is characterized by.*
- In addition to modifying the abstract, we have removed the misleading part of the definition of frailty.
- *Line 64: Please provide a citation to support this change in demographic trend.*
- We have added one new citation (Ref No.5).
- *Line 71: The end of this sentence should say “…were pre-frail.”*
- We modified it according to the comment (Line 72).
- *Line 76: This is an interesting citation to select. There are a number of more recent consensus statements regarding frailty.*
- We have changed to a newer citation (Ref No.7).
- *Line 79: I do not think it is apt to say that one would diagnose frailty. Frailty is not a disease and not a commonly identified outcome in clinical practice, thus it would be more appropriate to say ”…for frailty identification.”*
- We modified it according to the comment (Line 80).
- *Line 82-83: Accumulation of Deficits Models*
- We modified it according to the comment (Line 84).
- *Line 85: “Frailty assessment” instead of “judgement”*
- We modified it according to the comment (Line 86).
- *Lines 94-97: It is unclear what the authors are trying to state and the English here could use a thorough review.*
- *Line 93-94: The English in these two sentences needs to be reviewed. It is difficult to understand what this sentence is trying to present. Additionally, no citations are provided to support the statement made.*
- We decided to delete this part because we thought that this part was not important in describing the background of our research.
- *Line 98-99: Citation required for this statement.*
- *Line 100-101: Please provide citations associated with the statements made here.*
- We have added one new citation (Ref No.19).
- *Line 101-102: What reports have identified these factors? What factors were identified? Please provide citations here as well.*
- We have added a new citation and added factors that have been identified as improving factors from frailty (Ref No.20, Line 100-102).
- *Line 108: “…in improving frailty status”*
- We modified it according to the comment (Line 110).
- *Lines 108-111: This sentence needs to be rewritten for clarity.*
- We deleted some sentences and modified the wording (Line 110-112).

**Methods**

- *Line 142; …frailty assessment*
- We modified it according to the comment (Line 142).

**Results**

- *Fig 2: The English use here could use some review. “stayed robust”, “stayed pre-frail”, “remained frail”*
- We modified it according to the comment (Fig 2).

**Discussions**

- *Line 284: No citations provided to support this statement.*
- *Line 287: No citations provided to support this statement.*
  - We have added a new systematic review as a citation and modified the subsequent text to match the citation (Line 273-275).
- *Line 295: “No-frailty people”?*
  - We have corrected the term you pointed out (Line 284).
- *Line 312-317: A lot of this text was already included in the results section. Please make this more succinct and focused on discussion aspects.*
- We have removed the repetitive expressions and modified them to be more concise (Line 319-322).
- *Line 337-338: Unclear what this sentence is stating.*
- With the re-calculation of the recovery rate from frailty, we revised the discussion regarding the change from pre-frail to robust (Line 322-325).
- Line 342: Please provide citations to support this statement.
- We decided that this part was not important for the discussion and deleted it.

**Conclusions**

- *Again, these findings are stated quite strongly for an observational study. It should be clear that these findings are associations.*
- We have made general corrections to the content of this section (Line 376-380).
